# A disulfide-bridged single-stranded DNA nanotube for co-delivery of siRNA and chemotherapeutics in ovarian cancer therapy

**DOI:** 10.3389/fmed.2026.1798117

**Published:** 2026-05-28

**Authors:** Yan Luo, Aoxue Guo, Tianxin Sheng, Liting Han, Chuanqi Gu, Xin Li, Congzhou Chen, Xiaolong Shi

**Affiliations:** 1 Department of Obstetrics and Gynecology, Renmin Hospital of Wuhan University, Wuhan, China; 2 College of Information Science and Technology, Beijing University of Chemical Technology, Beijing, China; 3 School of Computer Science and Cyber Engineering, Guangzhou University, Guangzhou, China

**Keywords:** cisplatin resistance, disulfide bond, DNA nanotubes, ovarian cancer, P-glycoprotein, poly (ADP-ribose) polymerase 1, siRNA

## Abstract

**Introduction:**

Using DNA nanotechnology, we designed a disulfide-bridged single-stranded DNA nanotube (SS-DNT) functionalized with external aptamers for targeting, and encapsulating chemotherapeutic agents internally to induce apoptosis in cancer cells.

**Methods:**

The SS-DNT itself and the siRNA attachment points are linked via disulfide bonds, which can be fully cleaved in the intracellular glutathione (GSH) environment of tumors, thereby enhancing the release efficiency of the drug and siRNA. This SS-DNT offers a cost-effective synthesis process and high efficiency in payload release.

**Results:**

The SS-DNT exhibited efficient payload release, potent cytotoxicity, and significant tumor growth inhibition, with no detectable systemic toxicity in normal cells.

**Discussion:**

With its cost-effective synthesis, efficient siRNA and drug co-delivery, and demonstrated therapeutic efficacy, this DNA nanodevice represents a promising platform for precision cancer therapy.

## 1 Introduction

Small interfering RNA (siRNA) is involved in tumor initiation, progression, metastasis, and drug resistance via the RNA interference (RNAi) pathway (1). It specifically targets messenger RNA (mRNA) in cancer cells, resulting in gene silencing and inhibition of tumor-related protein synthesis (2). Currently, siRNA has been extensively explored in clinical cancer therapy. However, native siRNA cannot easily penetrate cells, and non-targeted siRNA release may cause systemic toxicity (3, 4). Therefore, siRNA is often conjugated with aptamers or encapsulated within nanocarriers to enhance targeted delivery (5).

Common siRNA nanodelivery systems include lipid nanoparticles (6), polymer-based carriers (7), inorganic nanoparticles (8), and DNA origami (9), all of which improve siRNA stability and promote cellular uptake. However, conventional siRNA delivery platforms still face significant limitations, including poor tissue specificity, premature payload release, and the risk of systemic toxicity (10). DNA-based nanocarriers offer exceptional structural programmability, enabling precise control over geometry and functionalization for the co-delivery of multiple therapeutic agents (11). They can be sequence-designed for the accurate loading and release of targeting ligands, siRNA, and other agents (12). Generally, DNA nanodelivery system is constructed using DNA origami technology (13).

DNA origami exhibits versatile programmability, enabling the design of delivery vehicles with precisely controlled sizes and shapes (11). A wide range of therapeutic agents—including doxorubicin (14), immunostimulatory nucleic acids (15), siRNAs (9, 16), antibodies, and enzymes (17)—can be loaded onto DNA nanocarriers through various incorporation strategies. These strategies include intercalation, Watson–Crick base pairing, and covalent conjugation. DNA origami nanostructures provide programmable architectures with internal docking sites or cavities that encapsulate therapeutic payloads, protecting both the cargo and the surrounding environment (11, 16). DNA origami-based delivery platforms have facilitated diverse precision therapeutic strategies, such as thrombin-targeted delivery (18), tumor-responsive treatment (19), virus-specific recognition (20), and co-delivery of immune modulators. Despite these advances, DNA origami-based delivery systems still encounter major challenges, including immunogenicity, high production cost, and suboptimal drug release efficiency (21, 22).

First, DNA origami structures exhibit potential immunogenicity due to their exogenous nucleic acid composition (23). As these architectures are constructed from hundreds of short oligonucleotides hybridized onto a scaffold strand, the excessive presence of short strands may induce immunotoxic effects. The immunogenic response elicited by these structures is strongly sequence-dependent (24). Second, the fabrication of DNA origami remains expensive; although nucleic acid synthesis costs have declined in recent years, synthesizing the hundreds of short staple strands required still incurs costs of several thousand U.S. dollars. More critically, the efficiency of drug release from DNA origami structures is profoundly affected by their macromolecular architecture (25). Common strategies employ DNA strand displacement (26) or chemically cleavable bonds—such as photosensitive azobenzene linkers (27) or disulfide bonds (28)—as structural locks or zippers. However, even upon successful unlocking, the intrinsic inertia of the large DNA framework often hinders efficient drug release, with therapeutic molecules remaining trapped within the origami structure.

**FIGURE 1:**
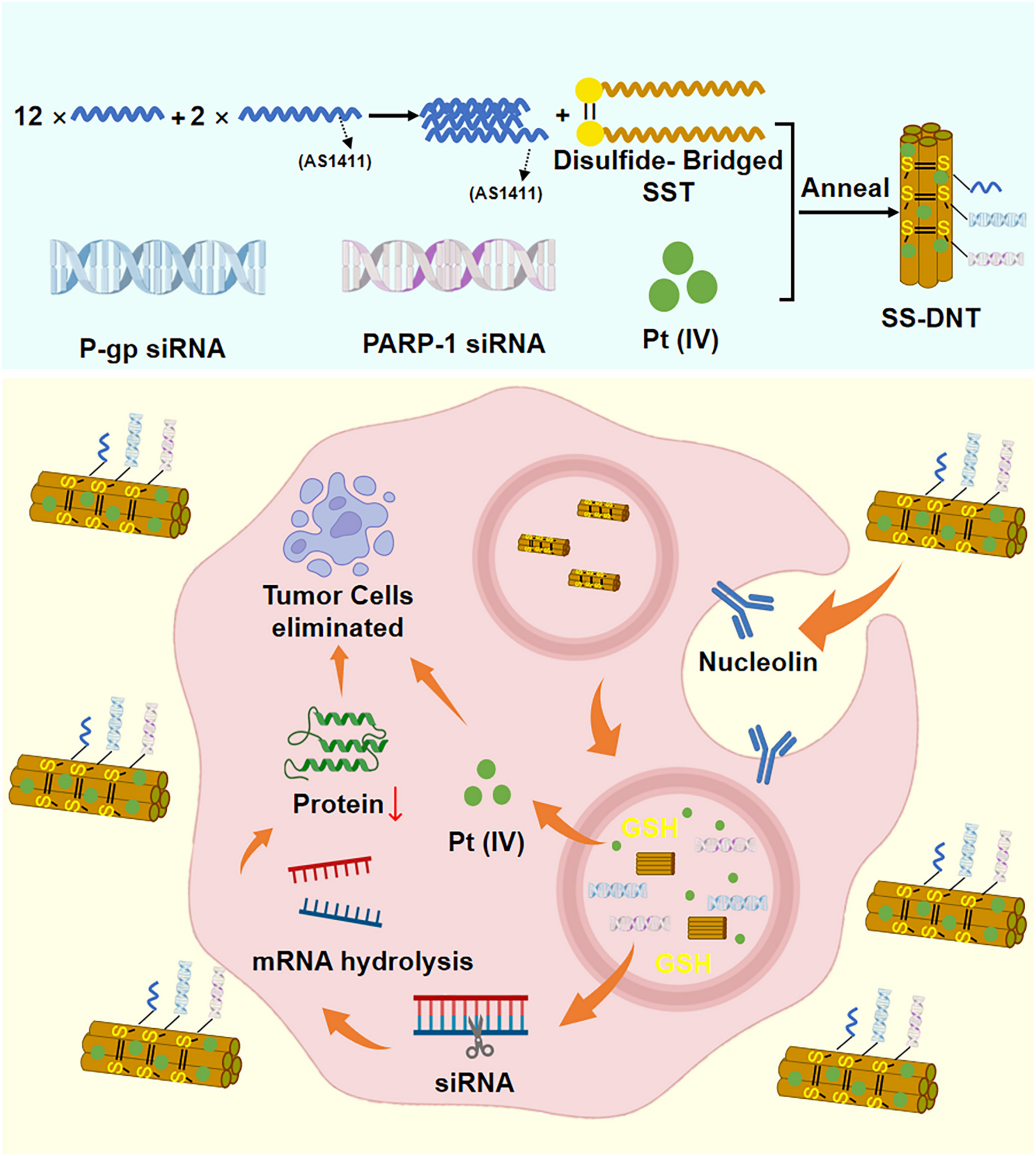
The design and mechanism of the study. The SS-DNT is a nanotube structure assembled from twelve short DNA strands and two ligand-modified strands, designed to co-deliver siRNA and platinum-based drugs. The SS-DNT system targets cancer cells via nucleolin recognition and is internalized through endocytosis. Within the tumor microenvironment characterized by elevated glutathione (GSH) levels, the SS-DNT disassembles, enabling the controlled release of both the siRNA and the chemotherapeutic agent.

In this study, we developed a novel programmable drug delivery system—SS—DNT—for ovarian cancer therapy. SS-DNT consists of 14 DNA single-stranded tiles (SSTs) (29), each SST contains a sulfenyl embedded in its central region to serve as a cleavable linking site. Our previous studies have demonstrated that the SST system can self-assemble into nanotubes with diameters ranging from 10 to 30 nm using two or three repeating SST motifs (30, 31). Here, we engineered the sticky ends of the SST units to co-load siRNAs targeting P-glycoprotein (P-gp) and poly (ADP-ribose) polymerase-1 (PARP-1). The inner cavity of SS-DNT was loaded with cisplatin prodrug Pt(IV). Additionally, the surface of the SS-DNT was functionalized with the targeting ligand AS1411 aptamer, facilitating specific recognition and uptake by cisplatin-resistant ovarian cancer cells. Notably, the linking sites of SS-DNT and the siRNA attachment points are all linked via disulfide bonds, enabling a complete cleavage and disassembling into intact single strands without leaving residual structures inside the cell, thereby enhancing vehicle release efficiency. Figure 1 illustrates the design and working mechanism of the study. The siRNA-Pt(IV)-aptamer co-loaded SS-DNT enabled localized gene silencing at the tumor site and demonstrated significant therapeutic efficacy against ovarian cancer.

## 2 Materials and methods

### 2.1 Synthesis of SS-DNT

The dry powder low-speed centrifuge contains 14 short chains and 1 long chain. A 20 μM solution is prepared using 1 × TAE buffer supplemented and 12.5 mM Mg^2+^. Twenty microliters (20μM) of the 20 μM short chain solution is taken and mixed to create a 280μL short chain mixture. The long chain solution is then combined with the short chain mixture in TAE buffer at varying molar ratios to achieve a final concentration of 100 nM. Synthesis is performed using the thermal annealing method. Following the mixing of siRNA, short chains, and long chains to a final concentration of 100 nM, siRNA is present at 12 times the molar amount of the short chain, while the long chain is present at 3 times the molar amount of the short chain. The synthesized SS-DNT are stored in a refrigerator at 4°C.

### 2.2 PAGE gel electrophoresis analysis

Thoroughly wash glass plates and other equipment. In a fume hood, sequentially mix 2 mL of 30% acrylamide-bisacrylamide (Acr-Bis) solution with 8 mL of 1 × TAE buffer and any additional components. Pour the resulting mixture into 1.0 mm glass plates, insert a comb, and allow polymerization to occur for 30 min at room temperature. Place the gel apparatus into an electrophoresis tank and add 1 × TAE buffer until the gel is fully submerged. Combine each DNA sample with 6 × DNA loading buffer, then load 10 μL of this mixture into each well. Conduct electrophoresis at 120 V for 90 min. Following electrophoresis, stain the gel in a solution of ultrapure water and nucleic acid dye (Biosharp, Beijing, China) for 20–30 min. Visualize the gel under ultraviolet light using a gel imaging system (Bio-Techne, San Jose, CA, United States).

### 2.3 Characterization of SS-DNT

A 10 μL sample was deposited onto a mica sheet, followed by 20 μL of 1 × TAE buffer, and allowed to adsorb for 15 min. The mica was then observed using an atomic force microscope (AFM) in Scan Analyzer-fluid mode (Multimode 8, Bruker).

For transmission electron microscopy (TEM) analysis, a copper grid was dipped in 5μL SS-DNT solution and allowed to sit for 10 min, followed by staining with 2μL of 3% uranyl acetate for 2 min. Excess liquid was blotted with filter paper, and the staining/blotting process was repeated. The grids were air-dried overnight and subsequently examined using a Talos F200C transmission electron microscope (Thermo Fisher Scientific, Waltham, MA, United States).

### 2.4 Serum and room temperature stability assays

A total of 10 μL of a 100 nM siPARP-1-loaded SS-DNT solution was combined with an equal volume of 167 μM TCEP solution and incubated for 30 min. Subsequently, 16 μL of the resulting mixture, along with 4 μL of 200 nM siPARP-1, 8 μL of 100 nM blank SS-DNT, and 8 μL of 100 nM siPARP-1-loaded SS-DNT, were subjected to analysis via PAGE gel electrophoresis.

### 2.5 Redox-responsive drug release via disulfide bond cleavage

A total of 10 μL of a 100 nM siPARP-1-loaded SS-DNT solution was combined with an equal volume of 167 μM TCEP solution and incubated for 30 min. Similarly, A total of 10 μL of a 100 nM siPARP-1-loaded SS-DNT solution was combined with an equal volume of 10 μM GSH solution and incubated for 8 h. Subsequently, 16μL of the resulting mixture, along with 4 μL of 200 nM siPARP-1, 8μL of 100 nM blank SS-DNT, and 8 μL of 100 nM siPARP-1-loaded SS-DNT, were analyzed using PAGE gel electrophoresis.

### 2.6 synthesis of pt (IV) compound

400 mg of cisplatin (cis-diamminedichloroplatinum(II)) was added to 15 mL of 30% H_2_O_2_ and stirred at 70°C for 5 h for oxidation. The solution was cooled overnight at 4°C, and yellowish- white crystals of c,c,t-diamminedichlorodihydroxoplatinum(IV) were collected by centrifugation.

Two hundred milligrams (0.6 mmol) of c,c,t-diamminedichlorodihydroxoplatinum(IV) were dissolved in 10 mL of dimethyl sulfoxide (DMSO). Concurrently, sixty milligrams (0.6 mmol) of succinic anhydride were dissolved in an additional 10 mL of DMSO. The two solutions were combined and stirred at room temperature for 12 h. The resulting product was lyophilized, washed with acetone and ether, and subsequently vacuum-dried to yield c,c,t-diamminedichlorodihydroxoplatinum(IV).

### 2.7 Synthesis and characterization of Pt(IV)-loaded SS-DNT

Synthesis of handle-Pt(IV) conjugate: A DNA strand modified with an amino group (NH_2_-CATCCCTAACTCTCA) was purchased from Sangon Biotech. The conjugation of Pt(IV) to the handle was performed via a standard NHS/EDC-mediated amide coupling reaction. NHS (1 M, 10 μL) and EDC (1 M, 10 μL) were mixed and incubated at 25°C for 15 minutes. Then, 10 μL of 0.1 M c,c,t-diamminedichlorodihydroxoplatinum(IV) in ddH_2_O was added and incubated for another 10 minutes. Finally, 10 μL of 25 μM amino-modified DNA handle was added, and the mixture was stirred at room temperature for 24 h.

Loading Pt(IV) onto SS-DNT: The 14 short strands, long strand, and handle-Pt(IV) strand were mixed at a ratio of 1:3:12 respectively, and annealed thermally to form Pt(IV)-loaded SS-DNT. The final products were characterized by DNA PAGE electrophoresis.

### 2.8 Cell culture

Human ovarian cancer cell line SKOV3, human cisplatin-resistant ovarian cancer cell line SKOV3/DDP, human embryonic lung cell line MRC-5, and mouse monocyte-macrophage cell line RAW 264.7 were all purchased from the China Center for Type Culture Collection (CCTCC, Wuhan, Hubei Province). SKOV3 and SKOV3/DDP cells were routinely cultured in RPMI-1640 medium supplemented with 10% fetal bovine serum and 1% penicillin-streptomycin mixture. MRC-5 and RAW 264.7 cells were routinely cultured in high-glucose Dulbecco’s Modified Eagle’s Medium (DMEM) containing 10% fetal bovine serum and 1% penicillin-streptomycin mixture. All cells were incubated in a constant temperature incubator at 37°C with 5% CO_2_.

### 2.9 Flow cytometry assay

Logarithmic-phase cells were centrifuged (300 × g, 5 min, 4°C), resuspended in 100 μL cold PBS, and incubated with primary antibody (5 μL, 1:100) for 30 min at 4°C in the dark. After two cold PBS washes (300 × g, 5 min), fluorochrome-conjugated secondary antibody (5 μL, 1:200) was added for 20 min at 4°C in the dark (indirect staining). Cells were washed, resuspended in 500 μL cold PBS, and analyzed on a flow cytometer with controls; and data processed via FlowJo (Version 10.8.1).

### 2.10 Cell apoptosis detection

Cells (1–10 × 10^5^), including those suspended in the culture supernatant, were harvested and washed twice with precooled phosphate-buffered saline (PBS) via centrifugation. The cells were then resuspended in 500μL of 1 × Binding Buffer, followed by the addition of 5 μL Annexin V-FITC and 10μL propidium iodide (PI; Liankebio, Hangzhou, China) to the sample. After gentle vortexing, the mixtures were incubated in the dark at room temperature for 5 min. Apoptotic cells were analyzed by flow cytometry using the FITC detection channel for Annexin V and the PE detection channel for PI.

### 2.11 Reactive oxygen species (ROS) detection

Cells from different treatment groups were incubated with 10 μM DCFH-DA (Beyotime Biotechnology, Shanghai, China) at 37°C for 30 min. After incubation, intracellular ROS levels were assessed by confocal laser scanning microscopy (CLSM; Nikon, Shanghai, China) and quantitatively analyzed by flow cytometry (CytoFlex, Beckman Coulter, IN, United States).

### 2.12 Intracellular uptake imaging by confocal microscopy

SKOV3/DDP cells were seeded in 35 mm glass-bottom dishes, cultured for 24 h, and incubated with pre-diluted, cargo-loaded SS-DNT. After washing and staining, imaging was performed using a Zeiss LSM880 confocal microscope. Data were processed with FlowJo software.

### 2.13 RT-qPCR

For the miRNA reverse transcription assay, the miRNA first-strand cDNA synthesis kit (tailing method) from Sangon Biotech was used. Quantitative reverse transcription polymerase chain reaction (qRT-PCR) was then performed to determine the relative expression levels of cellular P-glycoprotein (P-gp), poly (ADP-ribose) polymerase 1 (PARP-1), CD206, arginase-1 (Arg-1), tumor necrosis factor-alpha (TNF-α), and interleukin-1 beta (IL-1β), with the miRNA fluorescent quantitative PCR kit (dye method) supplied by Sangon Biotech.

### 2.14 Western blot

Proteins were extracted using a lysis buffer, followed by electrophoresis, membrane transfer, blocking, and incubation with primary and secondary antibodies. Detection was performed using a chemiluminescence imaging system, and quantitative analysis was conducted with ImageJ software.

### 2.15 Cytotoxicity assay

Seed the cells in a 96-well plate and incubate for 1 day. Following the drug intervention, add 10 μL of CCK-8 solution to each well and incubate for an additional hour. Measure the absorbance at 450 nm using a microplate reader (PerkinElmer, MA, United States).

### 2.16 Mitochondrial membrane potential assay (JC-1 staining)

Changes in mitochondrial membrane potential were detected using a mitochondrial membrane potential detection kit (Beyotime Biotechnology, Shanghai, China). The cell staining was observed under an inverted fluorescence microscope.

### 2.17 Cellular uptake pathway assay

To investigate the cellular uptake pathway of SS-DNT-dual-Pt(IV), SKOV3/DDP cells were treated with the following inhibitors at 37°C for 30 min: Amiloride, Chlorpromazine, and MβCD. Subsequently, SS-DNT-dual-Pt(IV) was added to the cells, followed by incubation for 24 h. Finally, the cells were harvested and the fluorescence intensity was detected by flow cytometry.

### 2.18 Statistical analysis

Data were analyzed using GraphPad Prism 8.0. All experiments were performed at least three times independently. Data are expressed as mean ± SD. Differences between groups were assessed by unpaired two-tailed *t*-tests, with *P* < 0.05 considered statistically significant.

## 3 Results

### 3.1 Construction and functional evaluation of disulfide-bond-stabilized SS-DNT

**FIGURE 2:**
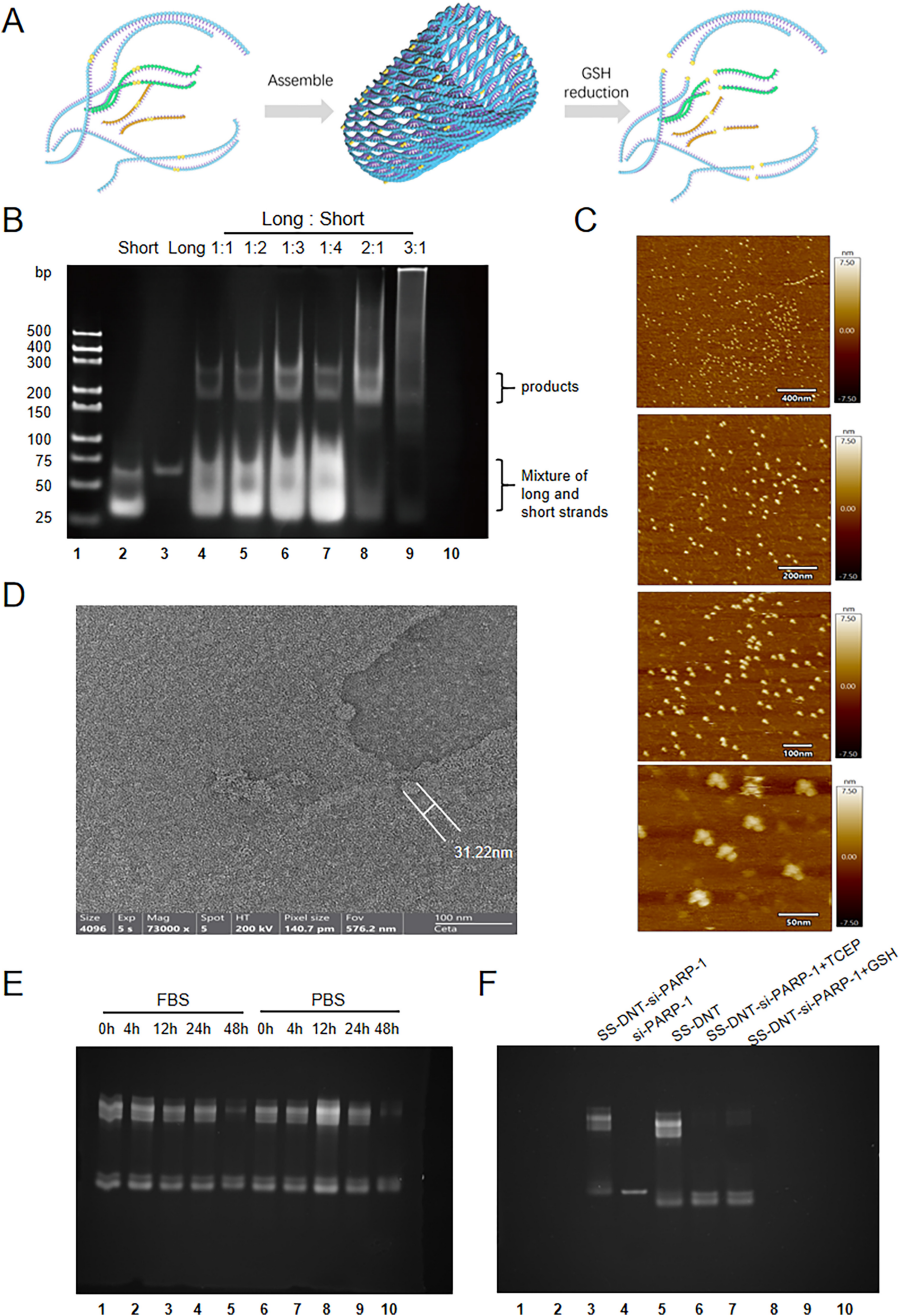
Characterization and functional evaluation of disulfide- bridged SS-DNT. **(A)** Schematic diagram of the six-helix bundle SS-DNT, these yellow dots represent double sulfur bonds and simulated 3D structure of the SS-DNT. **(B)** Native PAGE gel electrophoresis showing SS-DNT assembly with varying ratios of long and short strands;Lane1:Marker; Lane2:short strands; Lane3:long strands; Lane4-9:Structures formed by different ratios of long strands to short strands, with long-to-short strand ratios of 1:1, 1:2, 1:3, 1:4, 2:1, and 3:1. **(C)** Atomic force microscopy (AFM) images of SS-DNT at different magnifications; scale bars: 400 nm, 200 nm, 100 nm, and 50 nm, respectively. **(D)** Transmission electron microscopy (TEM) image of SS-DNT; scale bar: 100 nm. **(E)** Native PAGE gel electrophoresis of SS-DNT incubated in 37 °C serum and PBS at room temperature for various durations;Lane1-5: SS-DNT incubated in 37 °C serum for 0, 4, 12, 24, and 48 h. Lane 6–10: SS-DNT incubated in PBS for 0, 4, 12, 2, and 48 h. **(F)** Native PAGE gel showing siPARP-1-loaded SS-DNT before and after treatment with TCEP and GSH. Lane3: SS-DNT-si-PARP-1; Lane4: si-PARP-1; Lane 5: SS-DNT; Lane 6: SS-DNT-si-PARP-1 + TCEP; Lane 7: SS-DNT-si-PARP-1 + GSH.

A six-helix bundle SS-DNT was rationally designed using the honeycomb lattice framework in caDNAno,^1^ yielding a theoretical length of 34 nm and diameter of 5 nm (Supplementary Figure 1). The corresponding three-dimensional (3D) model is illustrated in Figure 2A. Native PAGE was employed to assess assembly efficiency by analyzing mixtures of long and short strands at different stoichiometric ratios (The detailed sequence information for the long and short strands is provided in Supplementary Figure 2). As shown in Figure 2B, increasing the proportion of short strands did not substantially promote nanotube formation, with most structures migrating between 200 and 300 bp. At a long-to-short strand ratio of 2:1, utilization of short strands markedly increased, suggesting enhanced assembly efficiency at this stoichiometry. Increasing the ratio further to 3:1 resulted in significantly elongated nanotubes, yielding high-molecular-weight assemblies (Supplementary Figure 3). Considering both strand utilization and structural integrity, a 2:1 ratio was determined to be optimal and was employed in all subsequent preparations (Supplementary Figures 4–6).

Atomic force microscopy (AFM) and transmission electron microscopy (TEM) were employed to characterize the morphology of the SS-DNT. As shown in Figure 2C, the tubes were distributed across the substrate surface, though local aggregation into star-like or clustered structures was observed instead of fully isolated linear tubes. The average length measured by AFM was ∼30.82 nm, closely matching the theoretical value. Similarly, TEM imaging (Figure 2D) showed that the SS-DNT assembled into dense ring-like or aggregated structures. Individual nanotubes measured ∼31.22 nm in length, consistent with AFM data and within acceptable deviation from the theoretical value.

Stability assays revealed that SS-DNT began to degrade after 24 h in serum-containing medium, with degradation progressively increasing in a time-dependent manner (Figure 2E). By contrast, incubation in PBS at room temperature resulted in minimal degradation over 24 h, indicating greater stability under non-enzymatic conditions.

Native PAGE was further employed to assess the siPARP-1 loading efficiency (Figure 2F). Compared to unloaded SS-DNT, annealing with siPARP-1 led to an upward band shift, indicating successful siRNA incorporation, which increased the hydrodynamic size and reduced electrophoretic mobility. Upon incubation with the reducing agent TCEP for 30 min, the SS-DNT band disappeared, and a new band appeared at the siPARP-1 position, indicating disulfide bond cleavage and efficient siRNA release under reductive conditions. Similarly, after incubation with 10 mM GSH for 8 h, the SS-DNT band almost disappeared. These findings confirm that disulfide-locked DNA nanotubes can efficiently load siRNA and enable redox-responsive release in lysosome-mimicking environments via glutathione-mediated disulfide cleavage.

### 3.2 Internalization and mechanism of action of siRNA-loaded ss-DNT

**FIGURE 3:**
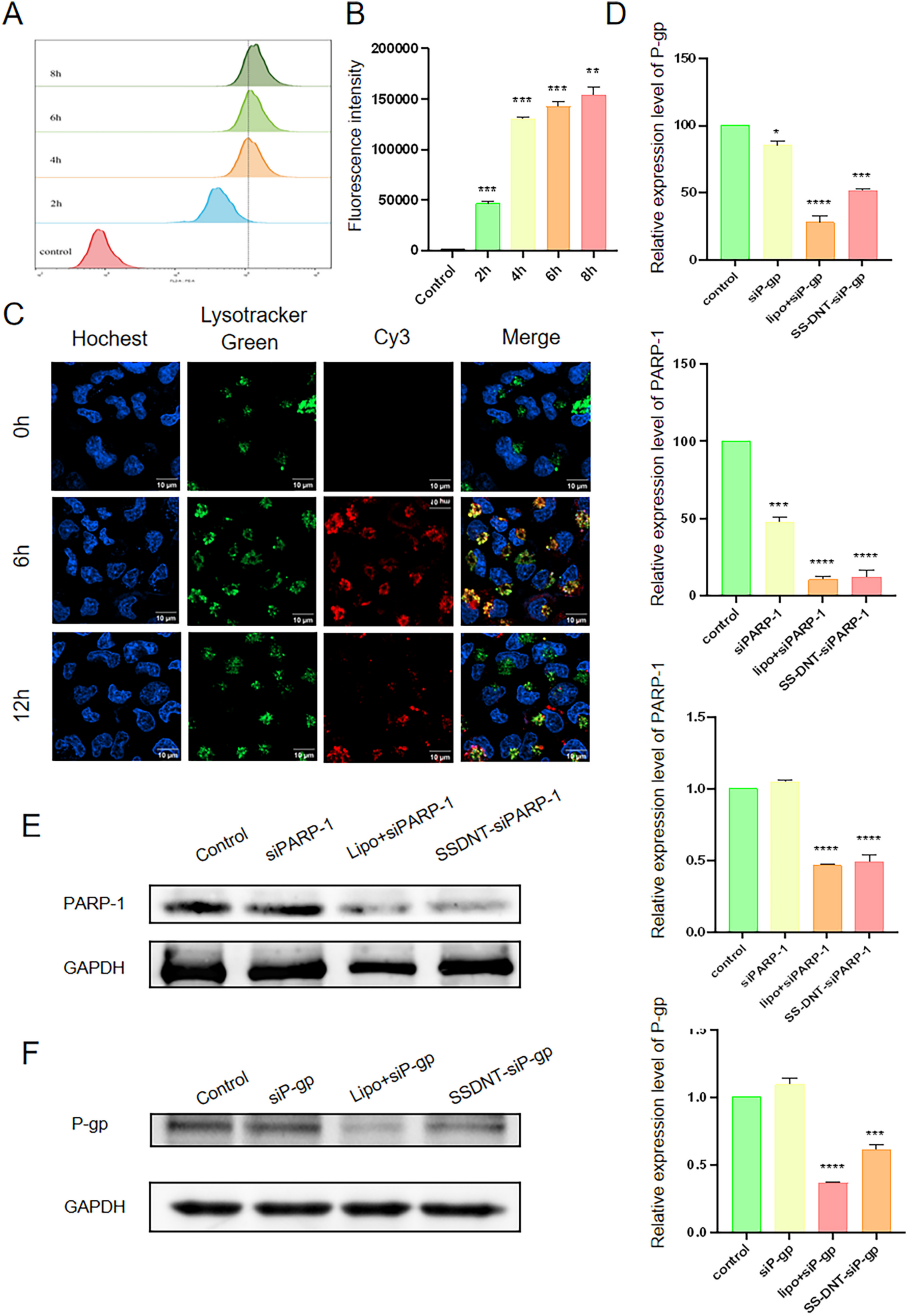
Cellular uptake, gene expression, and protein analysis of SS-DNT-mediated siRNA delivery. **(A)** Flow cytometry analysis of cellular uptake efficiency. **(B)** Quantification of Cy3 fluorescence intensity for evaluating siRNA delivery. **(C)** Lysosomal co-localization analysis of SS-DNT in SKOV3/DDP cells at 0, 6, and 12 h post-treatment. **(D)** Relative expression levels of P-glycoprotein (P-gp) and PARP-1 mRNA following different drug treatments. **(E)** Western blot analysis of PARP-1 protein expression and densitometric quantification across treatment groups. **(F)** Western blot analysis of P-gp protein expression and corresponding densitometric quantification across treatment groups. *p < 0.05, **p < 0.01, ***p < 0.001, ****p < 0.0001.

To quantitatively assess the cellular uptake efficiency of SS-DNT in SKOV3/DDP cells, flow cytometry was employed. This high-throughput, single-cell technique enables rapid and precise quantification of fluorescently labeled SS-DNT internalization, offering critical insights into their intracellular distribution and uptake dynamics. SS-DNT loaded with 100 nM Cy3-labeled handle strands were incubated with SKOV3/DDP cells for 2, 4, 6, or 8 h. Following incubation, cells were harvested and analyzed by flow cytometry. As shown in Figure 3A, Cy3 fluorescence intensity in SKOV3/DDP cells increased significantly after 2 h compared to the untreated control, indicating efficient cellular uptake of SS-DNT. Fluorescence intensity continued to rise with prolonged incubation, reaching a plateau at 4 h. These data suggest that most nanotube internalization occurred within the first 2 h and remained stable thereafter. Quantification of Cy3 fluorescence over time (Figure 3B) revealed significant differences from the control at all time points. These findings confirm the efficient cellular uptake of SS-DNT, providing a basis for their further application in siRNA and therapeutic cargo delivery.

Building on these findings, laser scanning confocal microscopy (LSCM) was used to examine the intracellular distribution of SS-DNT and their release and escape from lysosomes. As shown in Figure 3C, after 6 h of incubation with Cy3-labeled SS-DNT, nuclei were stained with Hoechst and lysosomes with LysoTracker Green. Intense cytoplasmic Cy3 fluorescence confirmed efficient internalization and lysosomal accumulation of the nanotubes. Comparison of confocal images at 6 and 12 h revealed a marked decrease in red-green fluorescence co-localization at 12 h (Table 1). These findings suggest that after 6 h, a subset of disulfide-crosslinked SS-DNT escapes from lysosomes, facilitating the release of encapsulated payloads into the cytosol. Collectively, the results confirm that these dual-functional nanocarriers exhibit efficient internalization and enable spatiotemporally regulated release of small-molecule therapeutics via pH-responsive lysosomal escape within 12 h. This sequential process—lysosomal accumulation, payload destabilization, and cytosolic translocation–ultimately enables targeted biological activity.

**TABLE 1:** Fluorescence co-localization analysis in SKOV3/DDP cells.

Incubation time (h)	Pearson’s *R*-value
6	0.59
12	0.05

Pearson’s *R* = 0.5–1 indicates positive co-localization; Pearson’s *R* = 0–0.5 indicates no significant co-localization.

We incubated SKOV3/DDP cells with SS-DNT loaded with siP-gp or siPARP-1 for 48 h, using lipofectamine-mediated siRNA transfection as a positive control and naked siRNA as an efficacy reference group. Cellular mRNA and protein were then extracted to assess gene silencing efficacy. As shown in Figure 3D, qPCR analysis revealed that naked siP-gp had minimal effect on P-gp mRNA levels, whereas naked siPARP-1 suppressed PARP-1 mRNA expression by approximately 50% relative to untreated controls. Lipofectamine-mediated siRNA transfection achieved ∼75% inhibition of P-gp mRNA and ∼90% inhibition of PARP-1 mRNA, consistent with established transfection efficiency. In contrast, SS-DNT-mediated delivery suppressed P-gp and PARP-1 mRNA by ∼50 and ∼88%, respectively, indicating effective siRNA delivery and target gene silencing by the SS-DNT.

Western blot analyses were subsequently conducted to assess whether the observed mRNA downregulation corresponded to a reduction in protein expression. As shown in Figures 3E,F, densitometric analysis revealed that SS-DNT-mediated siRNA delivery markedly suppressed the expression of both P-glycoprotein (P-gp) and poly (ADP-ribose) polymerase 1 (PARP-1), in contrast to the naked siRNA groups, which exhibited minimal changes. The degree of protein inhibition—approximately 50%—closely mirrored the mRNA knockdown efficiencies reported in Figure 3D. These results demonstrate the robust gene-silencing capability of SS-DNT and highlight their promise as targeted siRNA delivery vehicles for therapeutic applications.

### 3.3 Antitumor effects of siRNA-loaded SS-DNT

**FIGURE 4:**
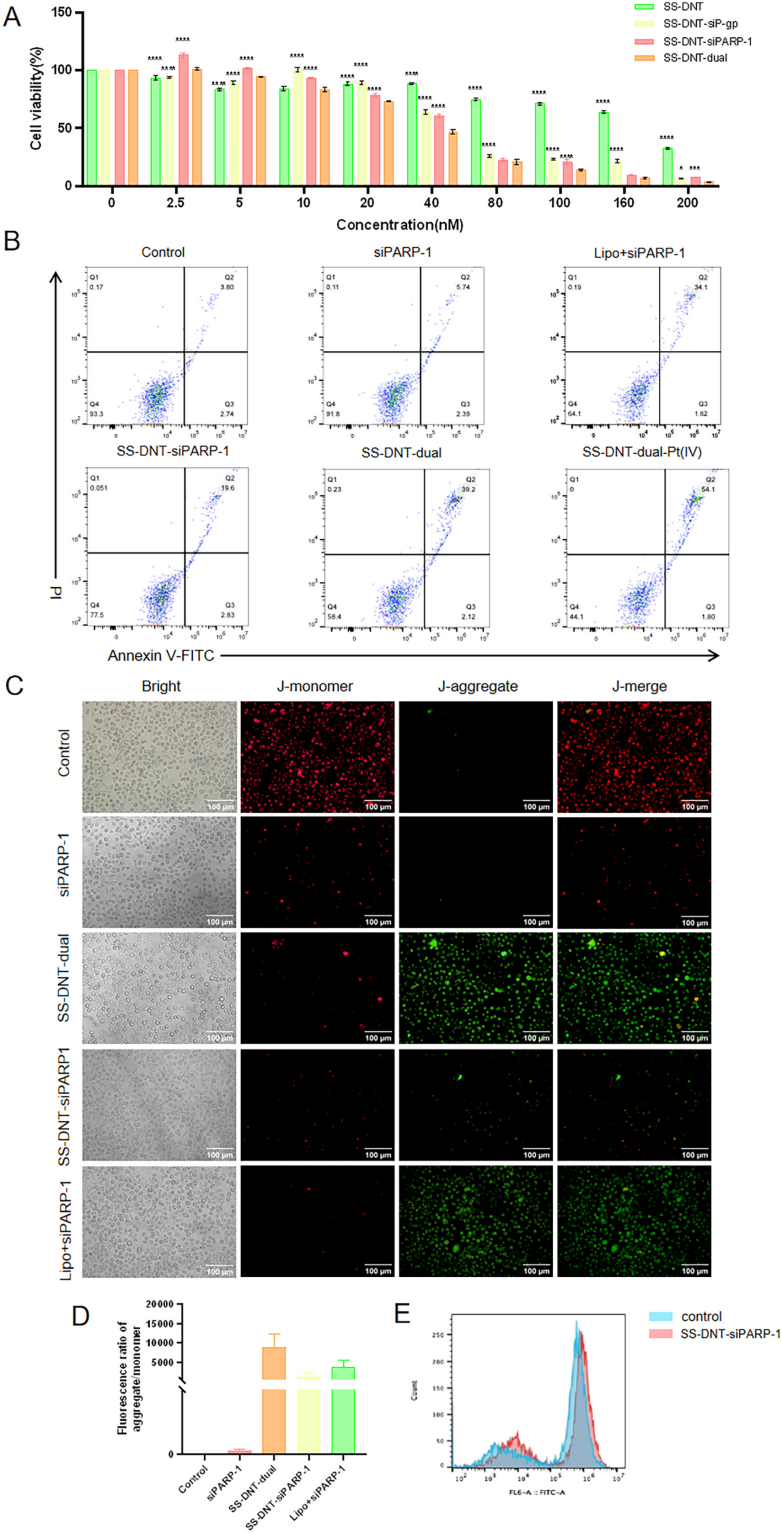
Cytotoxicity, apoptosis, and mitochondrial function of SS-DNT formulations. **(A)** Cytotoxicity analysis of cells treated with different formulations. **(B)** Apoptosis images of cells treated with various formulations. **(C)** JC-1 staining of cells treated with different formulations to evaluate mitochondrial membrane potential. **(D)** Quantitative analysis of JC-1 staining in cells treated with various formulations. **(E)** Intracellular reactive oxygen species (ROS) levels following treatment with SSDNT-siPARP-1. *p < 0.05, ***p < 0.001, ****p < 0.0001.

Given the role of PARP-1 in DNA repair, apoptosis, and mitochondrial dysfunction, we next examined whether PARP-1-targeting SS-DNT could modulate related cellular processes. Prior studies have shown that co-delivery of multiple siRNAs is more effective in suppressing tumor cell viability than single-siRNA treatments. Based on this rationale, we hypothesized that simultaneous silencing of P-glycoprotein (P-gp) and PARP-1 would elicit enhanced anticancer effects. To test this, we compared the cytotoxicity of SS-DNT delivering either single or dual siRNAs. As shown in Figure 4A, although the three treatment groups exhibited similar overall cytotoxicity profiles, dual-siRNA-loaded SS-DNT produced more pronounced inhibitory effects than their single-siRNA counterparts at equivalent concentrations. All three formulations exhibited half-maximal inhibitory concentration (IC_50_) values near 40 nM, with cytotoxicity increasing in a dose-dependent manner. These findings underscore the therapeutic potential of SS-DNT as efficient platforms for combinatorial siRNA delivery.

Flow cytometry was initially employed to assess the apoptotic effects of PARP-1-targeting SS-DNT in SKOV3/DDP cells. As shown in Figure 4B, the majority of cells in both the blank control and naked siPARP-1 groups remained viable, with fewer than 10% exhibiting apoptotic features. In contrast, treatment with SS-DNT loaded with siPARP-1 for 48 h induced apoptosis in 22.43% of cells, while the apoptosis induced by SSDNT-Dual is 41.32%, which exceeded that induced by Lipo+siPARP-1 (35.72%). Moreover, the apoptosis rate induced by SSDNT-dual-Pt(IV) reached 55.9%. These results demonstrate that disulfide-crosslinked SS-DNT effectively trigger apoptosis in tumor cells, thereby mediating cytotoxicity.

Mitochondrial membrane potential (ΔΨm) disruption is an early hallmark of apoptosis. To further elucidate the mechanisms of siPARP-1-induced cellular damage, we employed the JC-1 fluorescent probe to evaluate ΔΨm in SKOV3/DDP cells. As depicted in Figures 4C,D, SS-DNT treatment carrying siPARP-1 significantly reduced red fluorescence, indicative of intact ΔΨm, while increasing green fluorescence, indicative of mitochondrial depolarization and dysfunction. Furthermore, flow cytometric analysis revealed a pronounced elevation in intracellular reactive oxygen species (ROS) levels in siPARP-1 nanotube-treated cells relative to the blank control (Figure 4E). Collectively, these data demonstrate that SS-DNT-mediated delivery of siPARP-1 impairs mitochondrial function, induces oxidative stress, and promotes apoptosis, reinforcing the therapeutic potential of this delivery platform for targeted gene silencing.

### 3.4 Drug delivery potential and biocompatibility of SS-DNT

**FIGURE 5:**
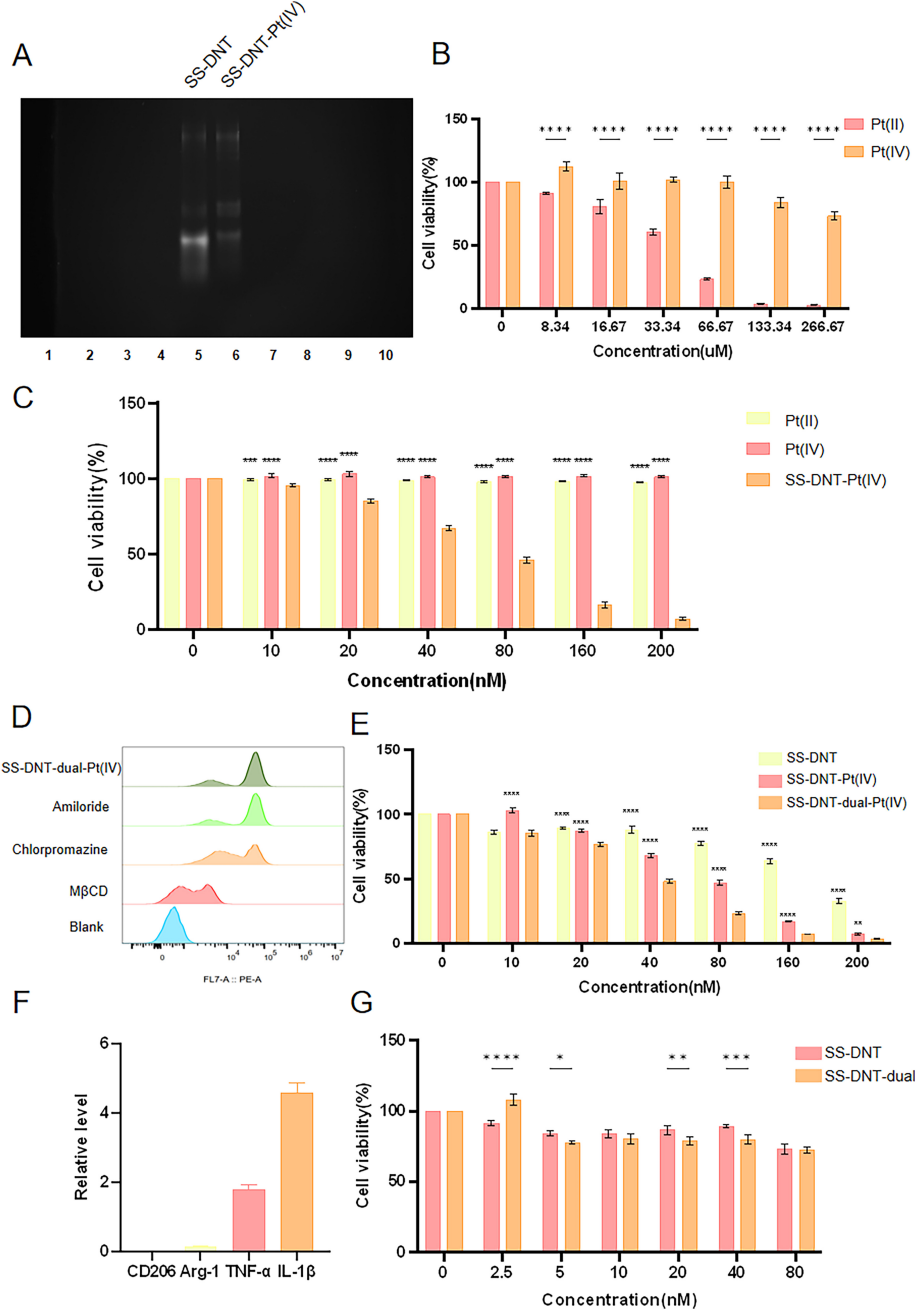
Cytotoxicity, combinatorial therapy efficacy, and biocompatibility of SS-DNT formulations. **(A)** DNA PAGE gel electrophoresis mobility assays of SS-DNT and SS-DNT-Pt(IV) for structural verification. Lane5:SS-DNT; Lane6: SS-DNT-Pt(IV). **(B)** Cytotoxicity comparison of cells treated with various drug combinations. **(C)** Cytotoxicity assessment of cells treated with distinct platinum-based formulations. **(D)** Cells were pretreated with Amiloride, Chlorpromazine, or MβCD, then incubated with SS-DNT-dual-Pt(IV). Fluorescence intensity was measured by flow cytometry to determine the uptake pathway. **(E)** Cytotoxicity profiling of cells treated with dual-siRNA/Pt(IV)-loaded SS-DNT at increasing concentrations. **(F)** Relative expression levels of immune cytokines in RAW 264.7 cells following SS-DNT treatment. **(G)** Cytotoxicity evaluation of MRC-5 cells treated with formulations at varying concentrations. *p < 0.05, **p < 0.01, ***p < 0.001, ****p < 0.0001.

As a novel drug delivery platform, SS-DNT shows significant potential for combination therapy with conventional chemotherapeutics. In the context of ovarian cancer chemotherapy, platinum-based drugs remain the first-line treatment option. DNA nanomedicines loaded with tetravalent platinum [Pt(IV)] can be locally converted to divalent platinum [Pt(II)] through reduction reactions within tumors, facilitating direct cancer cell destruction and enhancing chemosensitivity. According to our experimental protocol, divalent platinum [Pt(II)] was oxidized back to tetravalent platinum [Pt(IV)] via redox reactions. Subsequently, Pt(IV) was conjugated to SS-DNT building blocks using handle strands through thermal annealing, successfully resulting in the construction of Pt(IV)-loaded SS-DNT. The conjugation of Pt(IV) was confirmed by DNA PAGE gel electrophoresis mobility assays. As illustrated in Figure 5A, the bands of disulfide-crosslinked SS-DNT loaded with Pt(IV) exhibited an upward shift compared to those of unloaded SS-DNT, indicating successful Pt(IV) loading and stable nanotube formation.

To evaluate the cytotoxic effects of various drug combinations on SKOV3/DDP cells, we assessed the differential inhibition of cell proliferation by Pt(II), Pt(IV), Pt(IV)-loaded SS-DNT, and co-loaded with Pt(IV) and the two aforementioned siRNAs. Initially, we compared the cytotoxic efficacy of Pt(II) and Pt(IV) against SKOV3/DDP cells. As illustrated in Figure 5B, at a concentration of 33.34 μM, the Pt(II) group approached the IC_50_ value, with nearly complete cell death observed at 266.67 μM. In contrast, even at an 133.34 μM concentration, cell viability in the Pt(IV) group remained above 75%, indicating that Pt(IV) alone has difficulty penetrating tumor cell membranes to exert cytotoxic effects. Subsequently, we loaded platinum(IV) [Pt(IV)] onto SS-DNT and compared the cytotoxic effects of platinum(II) (Pt(II)), platinum(IV), and Pt(IV)-loaded SS-DNT. Based on the molar masses of Pt(II) and Pt(IV) (300.05 g/mol and 334.07 g/mol, respectively), all formulations were diluted to equivalent concentrations for comparison. As illustrated in Figure 5C, within the tested concentration range, both Pt(II) and Pt(IV) exhibited minimal cytotoxicity toward the cells. However, upon conjugation to SS-DNT, Pt(IV) demonstrated significantly enhanced cytotoxicity, inducing over 50% cell death at 80 nM and nearly complete tumor cell elimination at 200 nM. As illustrated in Figure 5D, SKOV3/DDP cells were treated with the following endocytosis inhibitors: Amiloride, Chlorpromazine, and MCD. Subsequently, SS-DNT-dual-Pt(IV) was added to the cells and incubated. Finally, the cells were harvested and the fluorescence intensity was detected by flow cytometry. The results indicated that the cellular uptake of SS-DNT-dual-Pt(IV) depends on lipid raft-mediated endocytosis. As shown in Figure 5E, the SS-DNT group co-loaded with both siRNAs and Pt(IV) achieved an IC50 value of approximately 40 nM, with cytotoxicity progressively increasing in relation to drug concentration. This finding suggests that the synergistic effect of siRNAs and Pt(IV) may enhance antitumor efficacy under high-concentration conditions. Collectively, these results demonstrate the significantly improved tumor-killing efficacy of Pt(IV)-loaded SS-DNT compared to traditional platinum-based drugs, thereby providing a new research direction for the development of multifunctional DNA nanomedicines and offering experimental evidence for optimizing combination strategies involving gene interference and chemotherapy.

To evaluate the *in vivo* immune response elicited by disulfide-crosslinked SS-DNT as exogenous agents, we assessed their immunogenicity by measuring the expression of key immune cytokines in mouse monocyte-macrophage RAW 264.7 cells. Quantitative PCR (qPCR) analysis was conducted to determine the relative expression levels of immune factors in RAW 264.7 cells following co-incubation with SS-DNT. As illustrated in Figure 5F, the expression of IL-1β increased by 4.5-fold, while TNF-α expression rose by 1.8-fold, indicating that the formulation enhances antitumor immune responses. Concurrently, the expression of CD206 and Arg-1 was significantly reduced, reflecting a decrease in the immunosuppressive state and a potential restriction of tumor growth. These findings demonstrate that SS-DNT activate pro-inflammatory pathways in RAW 264.7 cells, enhance pro-inflammatory responses within the tumor microenvironment, and diminish immunosuppressive states, collectively contributing to the inhibition of tumor progression.

To assess the safety of SS-DNT drug delivery systems in normal cells, we co-incubated pure SS-DNT and dual-siRNA-loaded SS-DNT with human embryonic lung MRC-5 cells for 48 h, evaluating cytotoxicity through CCK-8 cell viability assays. As illustrated in Figure 5G, both formulations demonstrated no significant toxicity toward MRC-5 cells at concentrations below 80 nM—the effective range for cell killing—indicating that SS-DNT exhibits high biocompatibility at therapeutic doses.

## 4 Discussion

In this study, we developed a disulfide-bridged, aptamer-functionalized SS-DNT drug delivery system and evaluated its ability in gene silencing, drug delivery, cytotoxicity induction, and immune modulation. Experimental results demonstrated that the SS-DNT enables efficient siRNA delivery and significantly enhances antitumor therapeutic efficacy. Owing to its low production cost and robust performance, this system holds considerable promise for future applications in precision cancer therapy.

In terms of gene silencing, SS-DNT successfully co-delivered siP-gp and siPARP-1, achieving efficient intracellular suppression of the corresponding target genes. qRT-PCR and Western blot analyses revealed significant downregulation of both mRNA and protein expression levels of P-gp and PARP-1 upon treatment with siRNA-loaded SS-DNT. Furthermore, apoptosis assays demonstrated that SS-DNT with siPARP-1 markedly induced tumor cell apoptosis. JC-1 fluorescence analysis indicated a pronounced reduction in mitochondrial membrane potential, accompanied by a substantial increase in intracellular reactive oxygen species (ROS) levels. These findings suggest that the siPARP-1-loaded SS-DNT trigger apoptosis in tumor cells by disrupting mitochondrial function.

Immunogenicity assays demonstrated that the SS-DNT effectively activated the expression of pro-inflammatory cytokines IL-1β and TNF-α in RAW264.7 macrophages, while simultaneously suppressing the expression of immunosuppressive markers CD206 and Arg-1. Cytotoxicity assays on human embryonic lung fibroblasts (MRC-5) revealed that the SS-DNT exhibited minimal toxicity toward normal cells within the therapeutically effective concentration range, indicating a favorable biosafety profile.

Although *in vivo* experiments are not included in this study, numerous reports have shown that DNA-based nanocarriers exhibit anti-tumor efficacy in mouse models (32–34). Given that our SS-DNT is a novel system, these literature findings provide supportive evidence for its potential *in vivo* effectiveness, warranting future animal studies.

In terms of structural design, we innovatively developed a DNA nanotube drug delivery system using a minimal set of single-stranded tiles (SSTs), which substantially reduced construction cost. Unlike DNA origami-based systems that typically require hundreds of short DNA strands and a long scaffold strand derived from phage plasmids, our design relies on only 14 short strands. This results in an estimated cost of merely one percent compared to DNA origami. Moreover, all connection points within the SS-DNT structure are formed via disulfide bonds, which are fully cleavable in the reductive intracellular environment of cancer cells, enabling complete structural disassembly. As a result, the drug release efficiency of SS-DNT is several-fold higher than that of conventional origami-based systems.

In summary, the disulfide-bridged SS-DNT siRNA/Chemotherapeutics delivery system offers an innovative strategy to overcome tumor drug resistance, enhance antitumor immunity, and optimize combinational therapy. Experimental results demonstrate that the SS-DNT exhibits strong therapeutic potential in gene silencing, chemotherapy synergy, and immunomodulation. Moreover, the SS-DNT platform combines high drug delivery efficiency with low manufacturing cost. We believe this multifunctional DNA nanocarrier holds significant promise for advancing cancer therapy.

## Data Availability

The original contributions presented in this study are included in the article/Supplementary material, further inquiries can be directed to the corresponding authors.

## Footnotes

1
http://cadnano.org
