# Supplementary material for: A disulfide-bridged single-stranded DNA nanotube for co-delivery of siRNA and chemotherapeutics in ovarian cancer therapy
Source: Front Med (Lausanne). 2026 May 28;13:1798117. doi: 10.3389/fmed.2026.1798117 (PMC13254165; doi:10.3389/fmed.2026.1798117)
Supplement: Supplementary file 1 [file Supplementary_file_1.docx]

Supplementary Material

## Supplementary Figures


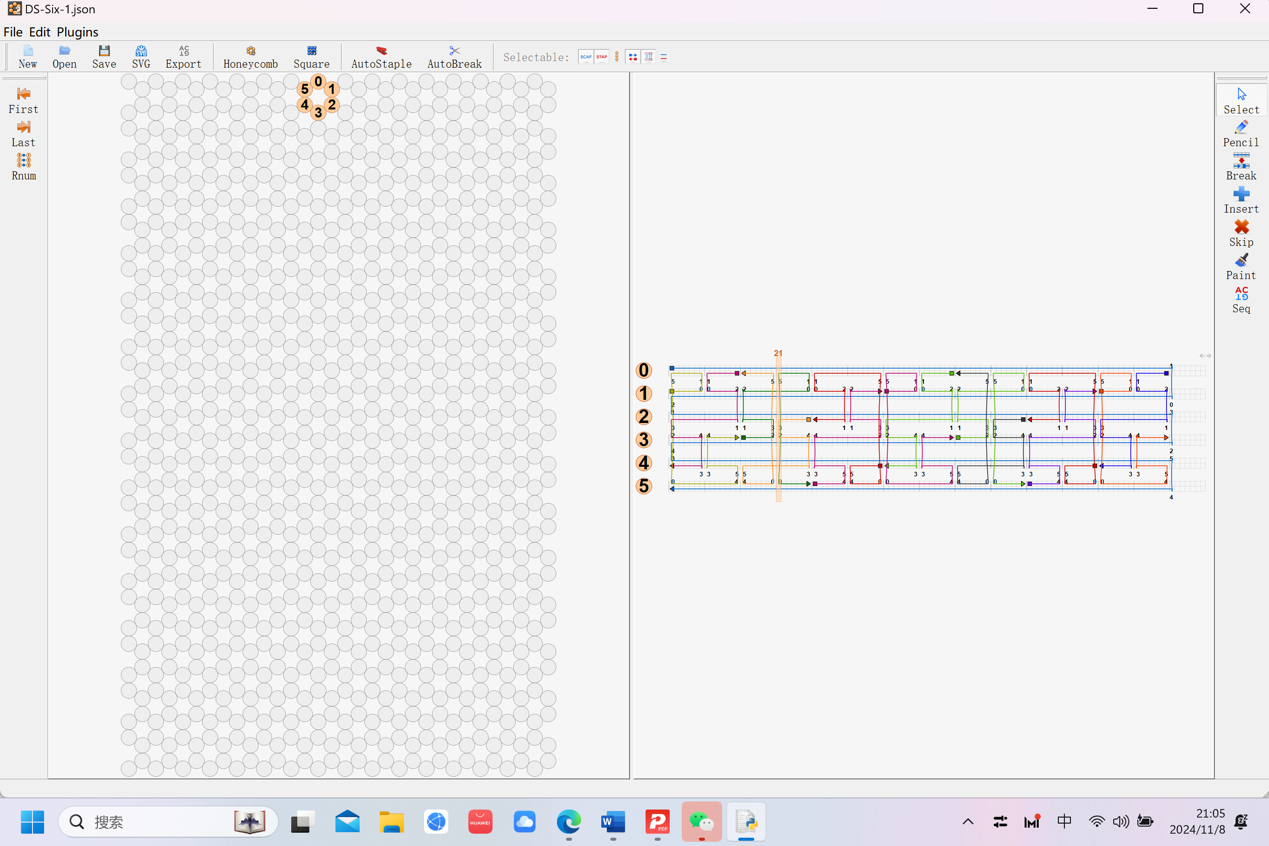

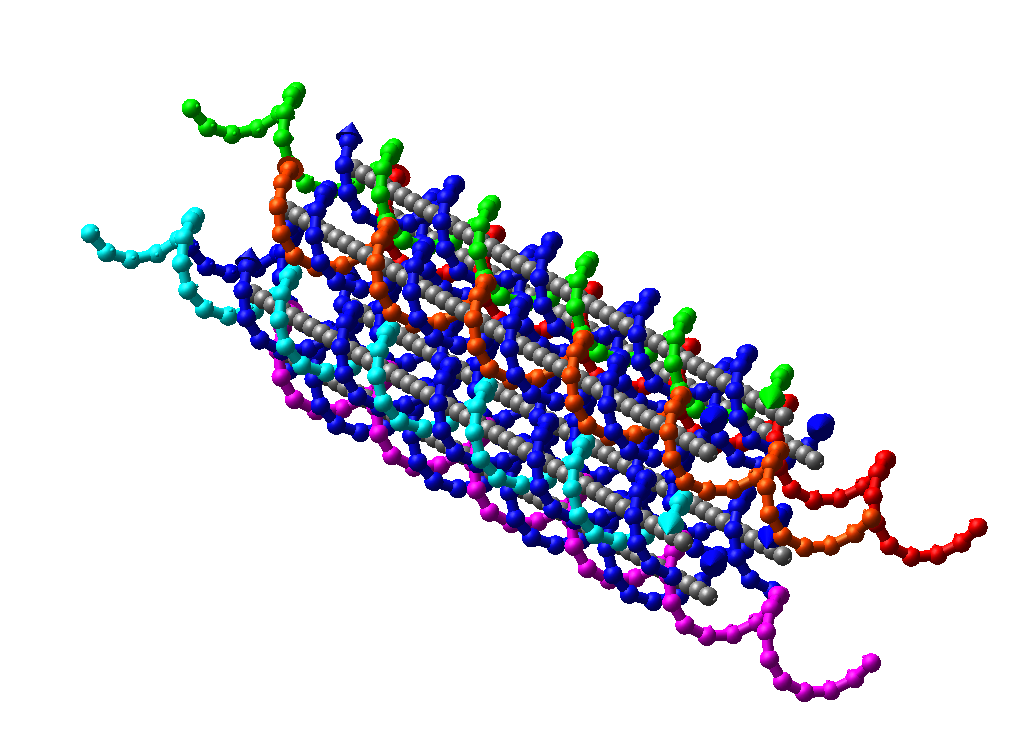


**Figure S1:** Schematic diagram of designing DNA nanotubes using caDNAno(Left); 3D structure diagram of DNA nanotubes(Right)

| Name | Sequence（5’-3’） |
| --- | --- |
| 0[13]-AS1411 | GCGTCGAGTCGAACGCGTCGAAATCAAAAGTATAGAATCAAA TGG TGG TGG TGG TTG TGG TGG TGG TGG |
| 1[84]-AS1411 | AGCGTCGTCGAACAAGCGTCGAAATCAAGTATAGGAAATCAA TGG TGG TGG TGG TTG TGG TGG TGG TGG |
| 0[97] | GTATAGGAAATCAAGTATAGGTCGAACAAGCGTCGTCGAACAtgagagttagggatg |
| 1[0] | AGTATAGAATCAAAAGTATAGGTCGAACGCGTCGAGTCGAACtgagagttagggatg |
| 1[42] | ATATCAGGGCCTAAATATCAGTGGCCTATATCAGTTGGCCTAtgagagttagggatg |
| 0[55] | TATCAGTTGGCCTATATCAGTGGCCTAAATATCAGGGCCTAAtgagagttagggatg |
| 2[27] | CTAATACAAGTGCGCTAATACGGACAAAAAAAGATGGACAAAtgagagttagggatg |
| 2[69] | CATGAAAAGTCCATCATGAAACCGTTAAAGCTCCACCGTTAAtgagagttagggatg |
| 3[14] | AAAAGATGGACAAAAAAAGATAAGTGCGCTAATACAAGTGCGtgagagttagggatg |
| 3[56] | AGCTCCACCGTTAAAGCTCCAAGTCCATCATGAAAAGTCCATtgagagttagggatg |
| 4[41] | GCTCCAAACCGTTAGCTCCAAGTCCATGTCATGAAGTCCATGtgagagttagggatg |
| 4[83] | AAAGATACGGACAAAAAGATAAGTGCGTGCTAATAAGTGCGTtgagagttagggatg |
| 5[28] | TCATGAAGTCCATGTCATGAAACCGTTAGCTCCAAACCGTTAtgagagttagggatg |
| 5[70] | GCTAATAAGTGCGTGCTAATACGGACAAAAAGATACGGACAAtgagagttagggatg |
| SST | TTTGATTTCGACGCTTTGTCCGTATTAGCATGGACTTGGAGCTTAGGCCtACTGATATTAACGG TTTCATGACGCACTTATCTTTTGTTCGACCTATACt |
| handle- Cy3 | Cy3 – CATCCCTAACTCTCA |

**Figure S2:** DNA and RNA sequences


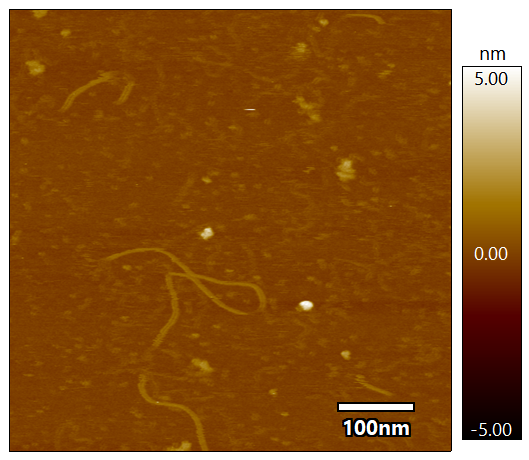

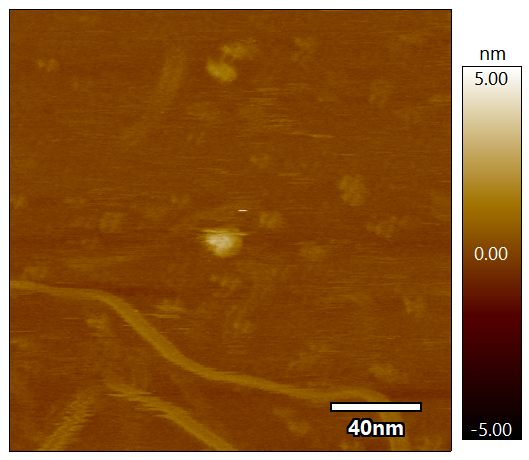


**Figure S3:** The 100 nm and 40 nm scale size of SS-DNT image with molar ratio of long to short strands 3:1.

When the molar ratio of long to short strands was set at 3:1, atomic force microscopy (AFM) results at scales of 100 nm and 40 nm, as shown in Figs. S3 and S4, revealed significant increases in nanotube length. Structures exceeding 50 nm were observed at the 100 nm scale, leading to the formation of high-molecular-weight aggregates. In certain regions, the winding of nanotubes was evident, resulting in bundle-like structures with diameters of approximately 10-15 nm. This observation suggests that a higher molar ratio of long strands may promote the emergence of topological defects during the assembly process.


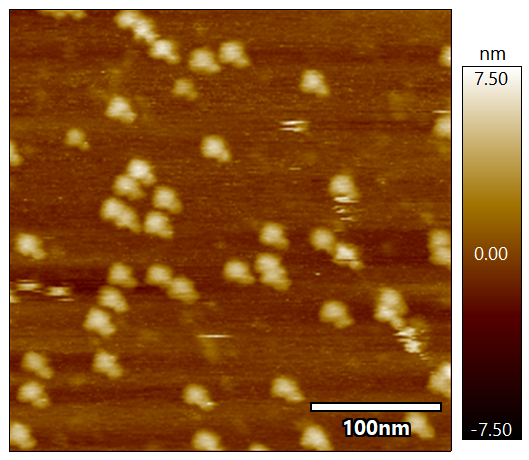

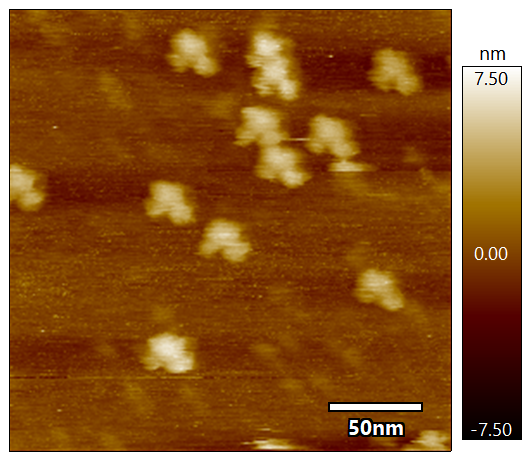


**Figure S4:** The 100 nm and 50 nm scale size of SS-DNT image with molar ratio of long to short strands 2:1 .

When the molar ratio of long to short strands was 2:1, the nanotubes exhibited a relatively uniform distribution across the substrate surface. The individual structures appeared as linear tubular shapes with diameters of approximately 5-7 nm, which closely aligned with the theoretically designed diameter of 5 nm. In the 100 nm scale image , the lengths of the nanotubes were predominantly concentrated in the range of 25-35 nm, with dense structures formed by the efficient assembly of short strands observed in localized areas. At the 50 nm scale, the surfaces of the nanotubes were smooth and devoid of significant protrusions, indicating that increased utilization of short strands contributes to enhanced structural integrity.


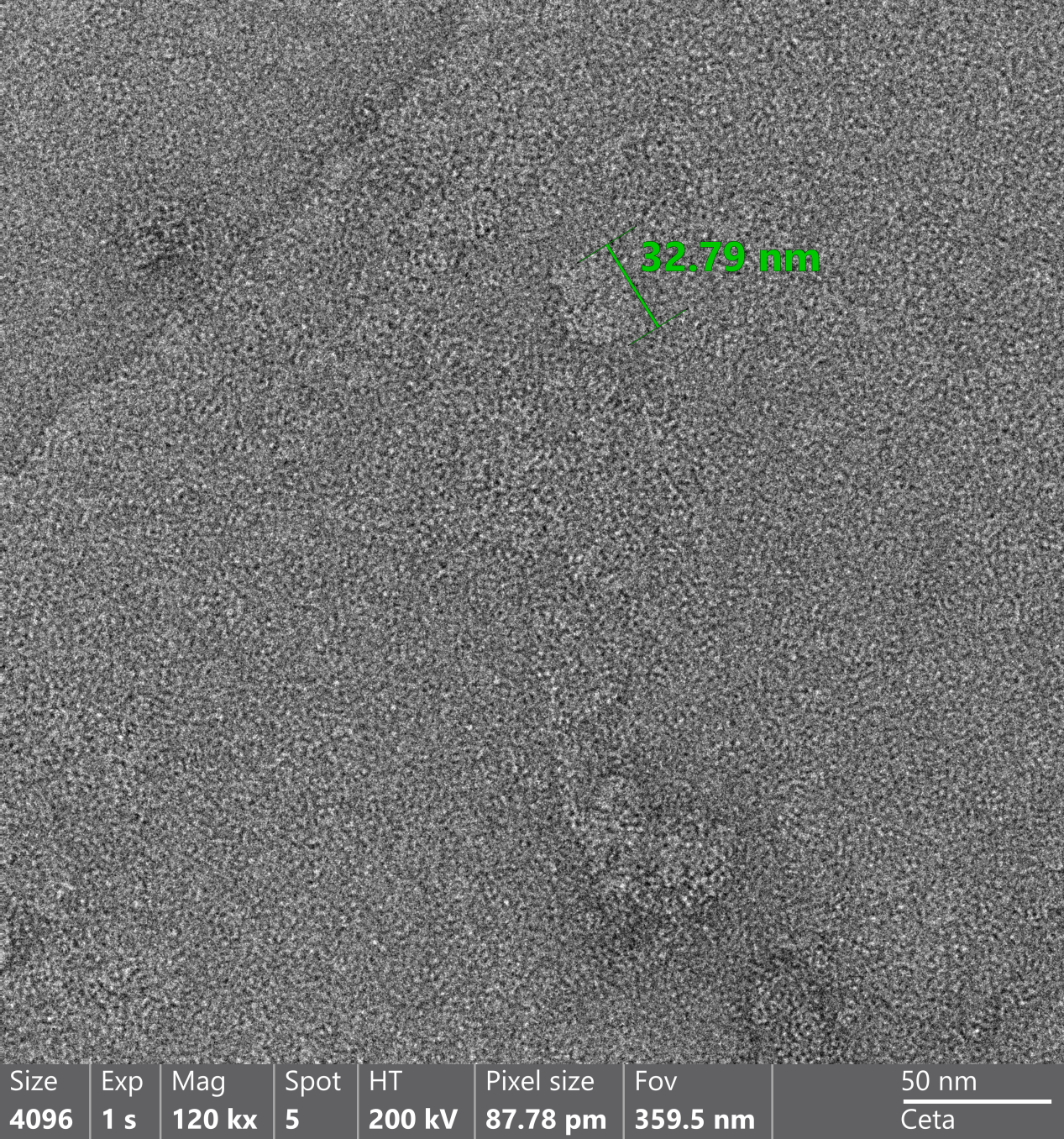


**Figure S5:** SS-DNT observed via transmission electron microscopy (TEM) at a scale of 50 nm.


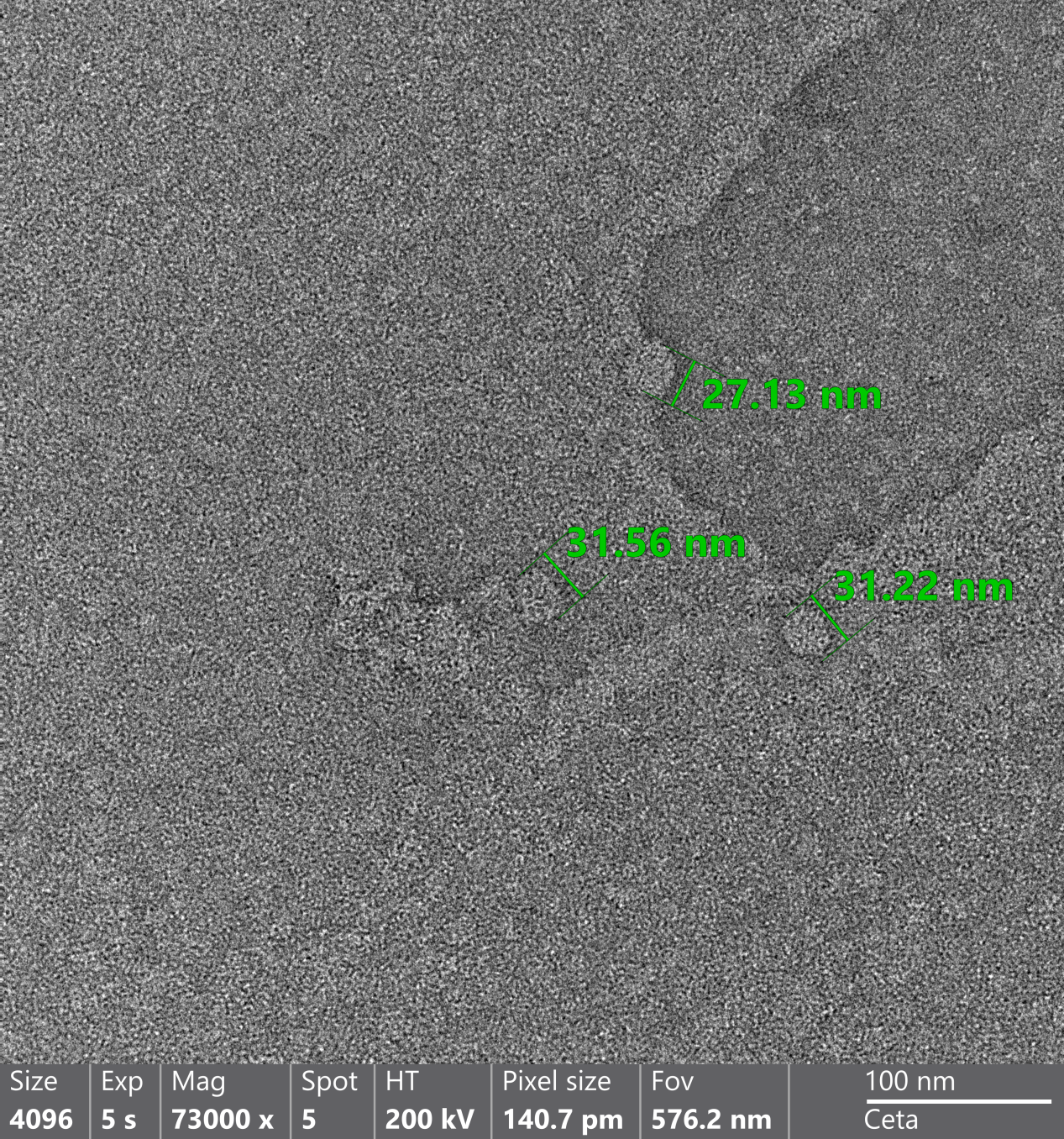


**Figure S6:** SS-DNT observed via transmission electron microscopy (TEM) at a scale of 100 nm.

Figure S5 illustrates the structures observed via transmission electron microscopy (TEM) at a scale of 50 nm, while Figure S6 presents the structures at a scale of 100 nm. The nanotubes displayed uniform ring-shaped or short rod-like morphologies, which align with the atomic force microscopy (AFM) results, thereby confirming the tubular structure of the six-helix bundle. The measured lengths of multiple nanotubes were 31.22 ± 1.89 nm (n = 15), with an error margin of less than 10% compared to the theoretical design value of 34 nm, thereby satisfying the precision requirements for self-assembled structures.
